# Bacterial endocarditis following COVID-19 infection: two case reports

**DOI:** 10.1186/s13256-023-03970-6

**Published:** 2023-06-16

**Authors:** Elham Barahimi, Sahar Defaee, Rahele Shokraei, MohammadHosein Sheybani-Arani, Ali Salimi Asl, Hossein Montazer Ghaem

**Affiliations:** 1 Infectious and Tropical Diseases Research Center, Hormozgan University of Medical Sciences, Bandar Abbas, Iran; 2 Faculty of Medicine, Hormozgan University of Medical Sciences, Bandar Abbas, Iran; 3 Student Research Committee, Faculty of Medicine, Hormozgan University of Medical Sciences, Bandar Abbas, Iran; 4 Department of Surgery, Faculty of Medicine, Hormozgan University of Medical Sciences, Bandar Abbas, Iran

**Keywords:** COVID-19, Secondary infection, Infective endocarditis, Tocilizumab

## Abstract

**Background:**

COVID-19, an emerging disease raised as a pandemic, urgently needed treatment choices. Some options have been confirmed as lifesaving treatments, but long-term complications must be clearly illustrated. Bacterial endocarditis is a less frequent disease among patients infected with SARS_COV_2 compared to other cardiac comorbidities in these patients. This case report discusses bacterial endocarditis as a potential adverse effect after administering tocilizumab, corticosteroids, and COVID-19 infection.

**Case presentation:**

In the first case, a 51-year-old Iranian female housewife was admitted to the hospital with fever, weakness, and monoarthritis symptoms. The second case is a 63-year-old Iranian woman who is a housewife admitted with weakness, shortness of breath, and extreme sweating. Both cases tested positive for Polymerase chain reaction (PCR) less than one month ago and were treated with tocilizumab and corticosteroid. Both patients were suspected of infective endocarditis. Methicillin-resistant *Staphylococcus aureus* (MRSA) was detected in the blood cultures of both patients. The diagnosis of endocarditis is confirmed for both cases. Cases are subjected to open-heart surgery, a mechanical valve is placed, and they are treated with medication. In subsequent visits, their condition was reported to be improving.

**Conclusion:**

Adjacent to cardiovascular inclusion as COVID-19 disease complications, secondary infection taken after the organisation of immunocompromising specialists can result in basic maladies and conditions counting infective endocarditis.

## Background

COVID-19 was identified in December 2019. The first infection was detected in a patient complaining of flu-like symptoms in Wuhan, China [1–3]. Respiratory involvement is the dominant feature of COVID-19 infection. It includes a broad spectrum of presentations, ranging from mild upper airway symptoms to acute respiratory distress syndrome (ARDS). Nevertheless, due to severe infection, multiorgan involvement is responsible for mortality besides ARDS [4, 5]. The COVID-19 infection viral phase is followed by the inflammatory phase, which results in multiorgan involvement. Macrophage activating syndrome (MAS) and increased levels of interleukin-6 (a pro-inflammatory cytokine) associated with cytokine storm in the inflammatory phase of COVID-19 infection are known to be involved in the pathogeneses of multiorgan damage. According to the latest COVID-19 treatment guidelines of the National Institutes of Health (NIH), using tocilizumab (an interleukin-6 (IL-6) receptor monoclonal blocking agent) combined with corticosteroid is one of the treatment options in severe COVID-19 infection. These agents also have approval from Food and Drug Administration (FDA) [6–8]. Although tocilizumab administration in COVID-19 is hypothesised that modulating IL-6 levels may reduce the duration or/and severity of COVID-19, suppression of the host immune response is considered a possible disadvantage of using this agent [9]. In addition, using corticosteroids as an immunomodulatory agent predisposes the patient to secondary infection. Here we are about to present two cases of bacterial endocarditis following tocilizumab administration and COVID-19 infection.

## Case report

### Case 1

The first case is a 51-year-old Iranian woman who is a housewife who presented with fever and pain in the left knee, which started about ten days ago, with oedema and warmth, limitation of active and passive movements, and no history of trauma. She complained about being febrile for the past week. She reported other symptoms, including cough, malaise, and dizziness. Her medical history included an episode of severe COVID-19 infection 4 weeks ago, confirmed by a positive positive Polymerase chain reaction (PCR) test and treated with tocilizumab, corticosteroid, and Remdesivir according to the latest COVID-19 treatment guidelines of NIH without any underlying disease. On examination, her conjunctiva was pale. The lung's auscultation was clear. Her heart’s auscultation manifested a third-grade holosystolic murmur which could be heard better alongside the lower left border of the sternum. The left knee is warm and edematous, and active and passive movements are limited. She was febrile with a temperature of 38.5 degrees Celsius, a blood pressure of 100/60 mm of mercury (mmHg), a respiratory rate of 16 breaths per minute, and an oxygen saturation of 97%. She had tachycardia, and her heart rate was 108 beats per minute (BPM). Her important lab data is in Table 1. Due to fever and monoarthritis, and suspicion of septic arthritis, arthrocentesis was performed for the patient. Then she was admitted to the ward and was started on meropenem 1 g via intravenous line every 8 hour and vancomycin 1 g via intravenous line every 12 hour. Methicillin-resistant *Staphylococcus aureus* (MRSA) was detected in blood cultures, and WBC = 35,000 and PMN = 92% were reported according to knee arthrocentesis.

**Table 1:** Laboratory data of cases

Lab data	Case 1	Case 2
WBC (10^9^/L)	16.2	12.2
Hb (g/dL)	9.9	8.5
MCV (fL)	71.4	80.4
PLT (10^3^/µL)	195	160
Creatinine (mg/dL)	1	1.2
Urea (mg/dL)	29	40
LDH (U/L)	548	606
SGOT (U/L)	44	41
SGPT (U/L)	49	40
Alp (U/L)	448	271
CRP (mg/dL)	65	78.7
Na (mg/dL)	135	138
K (mg/dL)	3.6	4.2

WBC: White Blood Cell Count, Hb: Hemoglobin, MCV: Mean Corpuscular Volume, PLT: Platelet Count, LDH: Lactate Dehydrogenase, SGOT: Serum Glutamic-Oxaloacetic Transaminase, SGPT: Serum Glutamic-Pyruvic Transaminase, Alp: Alkaline Phosphatase, CRP: C-Reactive Protein, Na^+^: Sodium Ion, K^+^: Potassium Ion

**Fig. 1:**
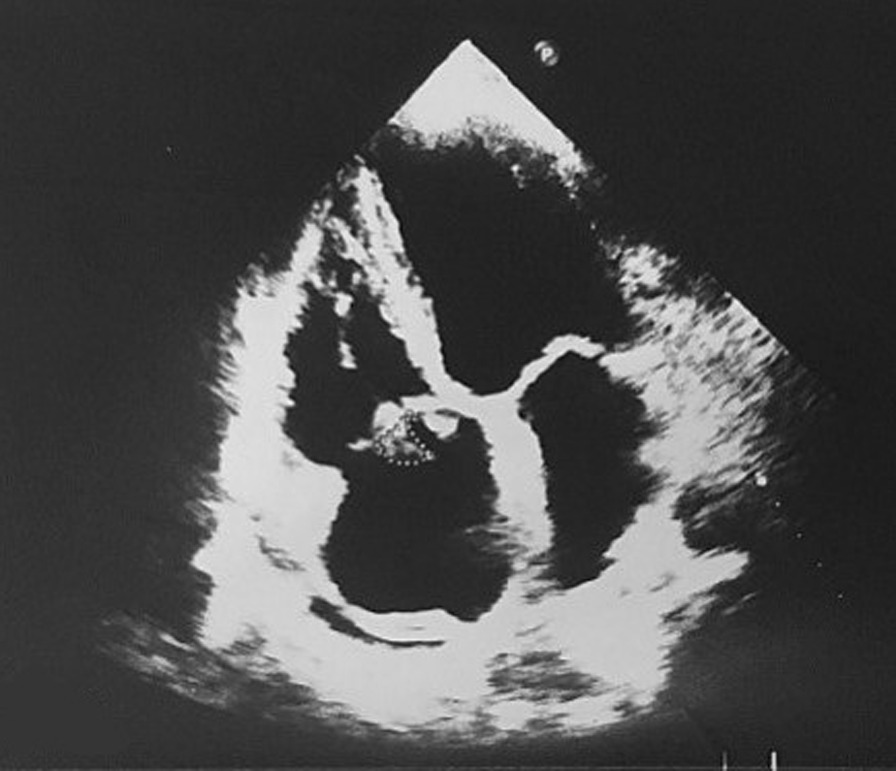
Highly mobile echogenicity on the anterior and septal leaflet tricuspid valve replacement with protruding to right ventricle cavity in favour of vegetation

**Fig. 2:**
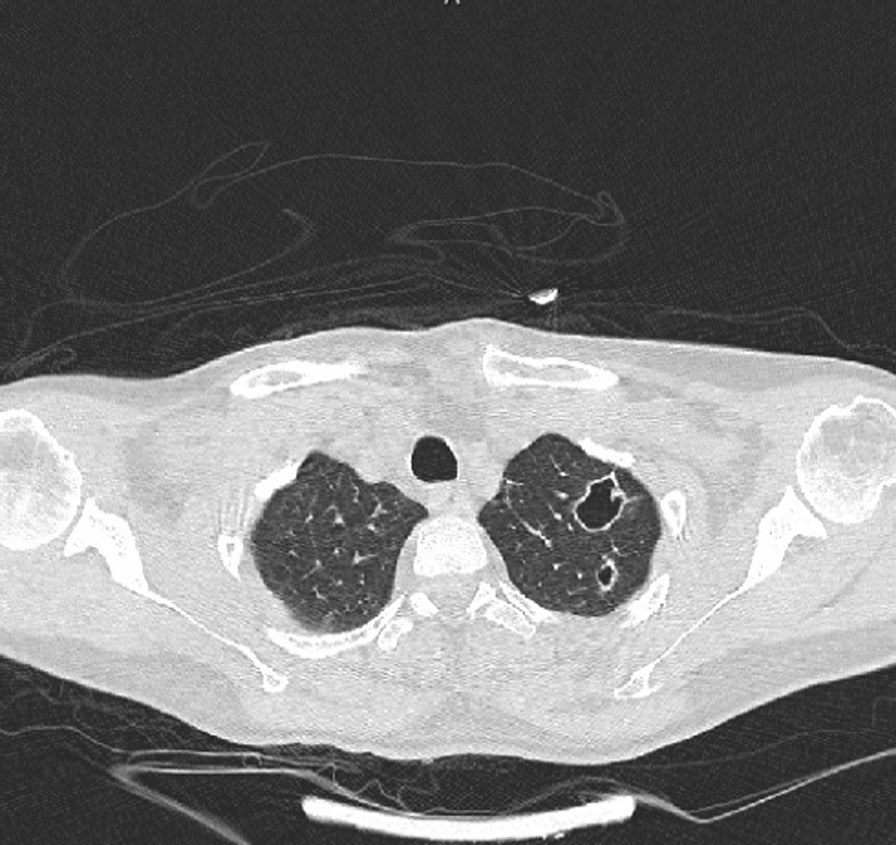
There are several nodules in both lower lobes; two cavitated nodule is also seen in the right lower lobe; one of them is subpleural with a feeding vessel sign

**Fig. 3:**
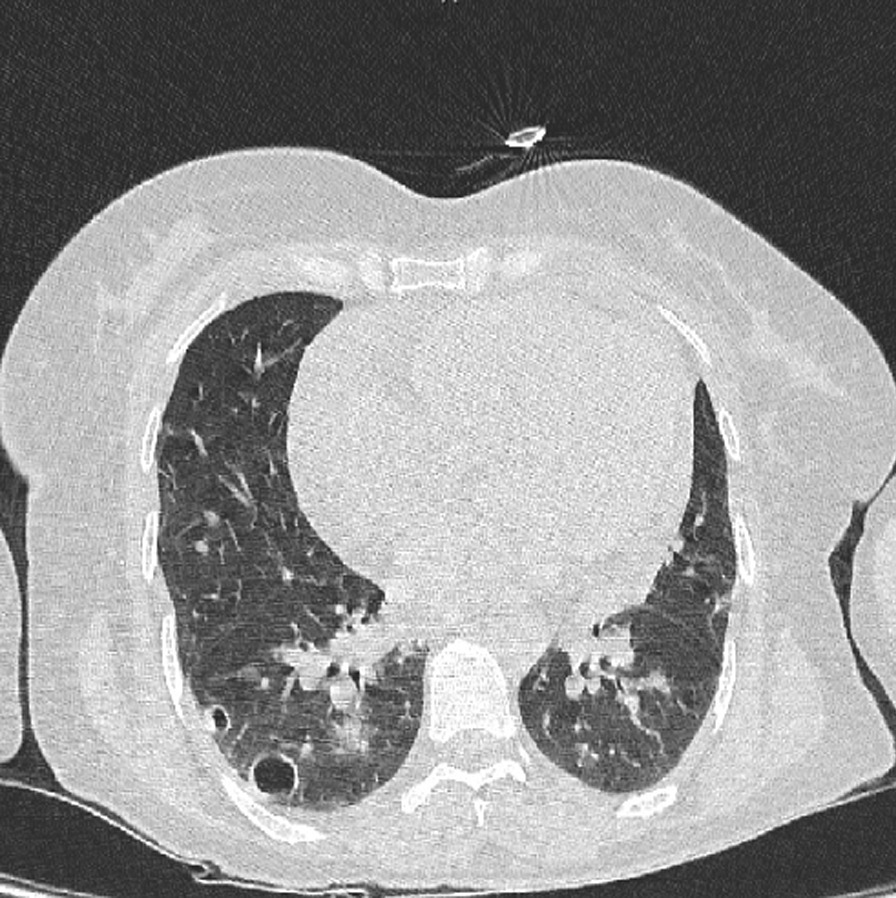
There are several nodules in both lower lobes; two cavitated nodule is also seen in the right lower lobe; one of them is subpleural with a feeding vessel sign

According to MRSA positive blood culture, with a probability of endocarditis considering the murmur, the case was consulted with a cardiologist who found highly mobile echogenicity on the anterior and septal leaflet with protruding to the right ventricle (RV) cavity in favour of vegetation, induced severe tricuspid regurgitation (TR) with tricuspid regurgitation gradient (TRG) of 17 mmhg, in addition to mild to moderate pulmonary insufficiency (PI) and mild pericardial effusion especially in the left posterior ventricle (LV) with systolic pulmonary pressure (SPAP) of 20–22 mmHg, via trans thoracic echocardiography Cardiologist decided to do an angiography on coronary arteries, which was expected and suggested abdominopelvic sonography and carotid arteries doppler sonography. There was no emboly nor flow decrease in carotid arteries detected by Doppler sonography and no significant abnormal finding in abdominopelvic sonography other than an accessory spleen measured 17.4 mm in diameter (Fig. 1). Based on the COVID-19 infection history and prolonged cough and fever, a respiratory consult and spiral chest computed tomography (CT) scan were done. In the Chest (CT) scan Tree in bud pattern, multi-lobar cavitation, nodule, and air bronchogram consolidation were seen (Figs. 2 and 3). To hypermobility of vegetation and prolonged fever (tricuspid valve replacement (TVR) indication), the patient's condition was consulted with a cardio surgeon who decided on doing a TVR during open-heart surgery. The valve was placed with a 31-size saint Jude mechanical valve. The result of the surgery and recovery period was excellent. After recovering from the surgery and significantly improving her clinical condition, she was discharged. Alarm signs and follow-up refer date was notified. Two weeks after discharge, the patient's cough, shortness of breath, and limitations of active and passive movements were utterly improved.

Multiple irregular cystic lesions exist in both long fields; some show cavitation. Also, some subpleural nodules with cavitation show feeding vessel signs.

### Case 2

**Fig. 4:**
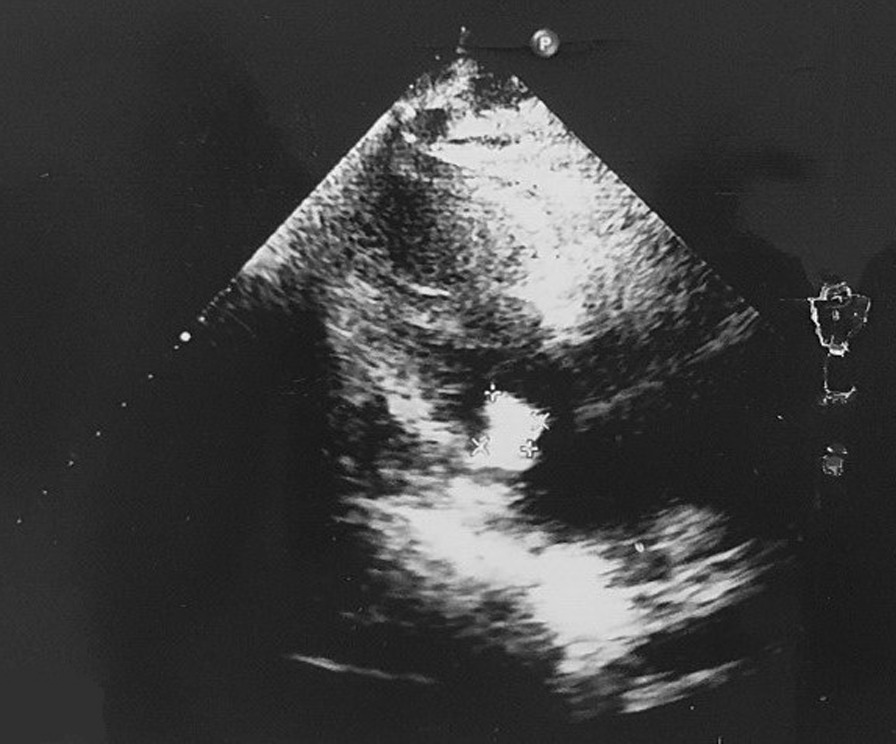
Shaggy appearance echogenicity with multiple highly mobile particles in favour vegetation on the posterior mitral leaflet

**Fig. 5:**
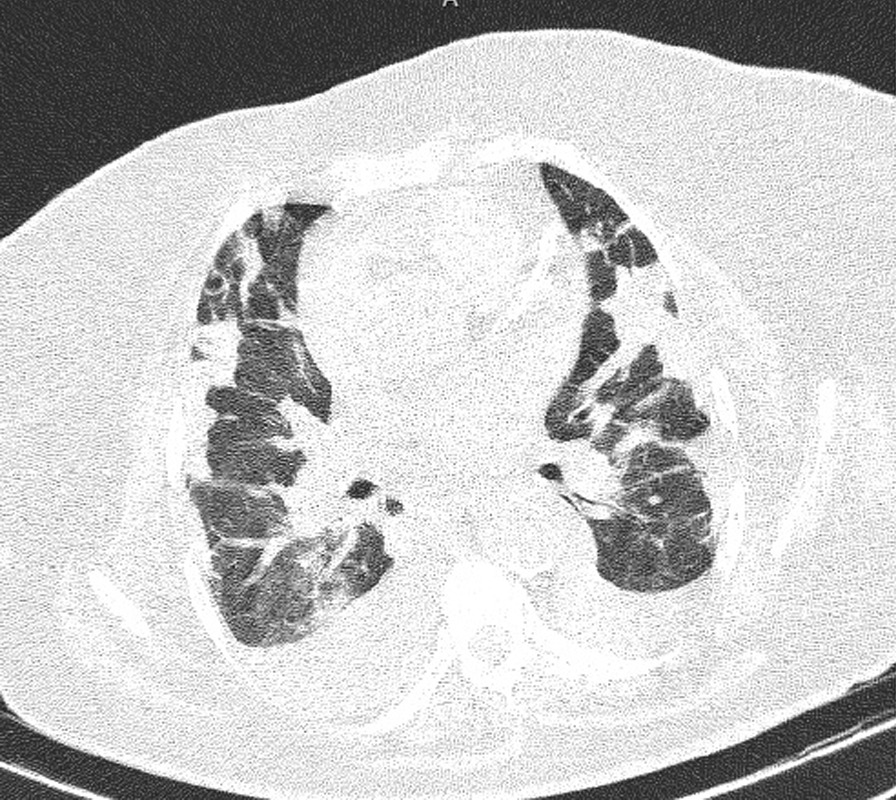
There are multiple patchy consolidations and ground-glass opacities in both lower lobes with peribronchial thickening)

The second case is a 63-year-old Iranian housewife who presented with weakness, headache, dyspnea, and extreme sweating. In her past medical history, she was a known case of Diabetes mellitus (DM) type II and hypertension (HTN). She reported her previous hospitalisation about 3 weeks ago due to severe COVID-19 infection, confirmed with PCR positive test, which was treated according to NIH's latest COVID-19 treatment guidelines. Her drug history included taking metformin 500 mg 1 tablet Bd and valsartan 80 mg 1 tablet every night. On examination, she had dyspnea but no respiratory distress. Her vital signs were typical: a temperature of 37.8 degrees Celsius, a heart rate of 95 bpm, normal blood pressure of 120/80, a respiratory rate of 25 breaths per minute, and oxygen saturation of 94%. Her conjunctiva was pale. No acanthosis nigricans was seen. Her lung’s auscultation manifested fine rale at the bottom on both sides. Her abdominal examination was normal, without any tenderness or abnormal findings. She had no abnormal findings in her extremities. Her nails had normal colour without clubbing. Her pulses were full and symmetric. Her Body Mass Index (BMI) was counted at about 33 kg per mitre to the second power (kg/m × m). Her important lab data is in Table 1. She was admitted to the ward with a COVID-19 infection complicated with hyperglycemia. After receiving conservative treatment and insulin administration, her blood sugar elevation was controlled. Her sweating and malaise were handled, and all symptoms were relieved after a week. However, the patient’s fever started again. We reevaluated the patient and detected a third-grade holosystolic murmur. According to auscultation findings and prolonged fever, endocarditis was suspected, and a cardiology consult was done. A spiral chest CT scan and echocardiography were ordered. Trans thoracic echocardiography was done, and vegetation or clot measured 17 mm × 8 mm on the anterior leaflet of the mitral valve accompanied by mild mitral regurgitation (MR) was depicted (Fig. 4). The chest CT scan showed multiple patchy consolidation and ground-glass opacities in the lower lobes with peribronchial thickening (Fig. 5).

Two blood cultures have been obtained. She was started on empiric antibiotic therapy, including meropenem 1 g via intravenous line every 8 hour and vancomycin 1 g via intravenous line every 12 hour. Based on MRSA detection in blood culture, the initial regimen was de-escalated. Second, follow-up echocardiography depicted 15.9 mm × 10.2 mm shaggy appearance echogenicity with multiple highly mobile particles in favour of vegetation. Moderate MR was detected, which had an eccentric jet toward the interatrial septum (IAS) seems to be a systolic flow reversal in two pulmonary veins. The cardiologist sentenced that although the vegetation density was increased. It was not likely to send embolies, according to degeneration of the mitral valve that was illustrated and moderate MR, which led to severe heart failure. The patient was a candidate for mitral valve replacement (MVR) surgery. After finishing an entire course of antibiotic therapy, the patient left the hospital against medical advice (PLHAMA). A week later, she came back with exacerbated symptoms. New echocardiography was done, illustrating more mitral valve degeneration than the former echocardiography and stronger MR. The cardiac surgeon consulted the case, and MVR via open-heart surgery was done. After recovering from the surgery, her clinical condition improved. She was discharged with notifying alarm signs and a follow-up refer date. Three weeks after discharge, the patient's shortness of breath and weakness improved entirely.

## Discussion

Here we presented two cases of bacterial endocarditis in patients with a history of COVID-19 infection treated by tocilizumab combined with corticosteroid and Remdesivir. COVID-19 infection's main symptoms are related to respiratory system involvement, including a broad spectrum of presentations, ranging from mild upper airway symptoms to ARDS [4, 5]. However, multiorgan involvement is a consequence of severe infection, which is responsible for mortality besides ARDS. one of the rising considerable organ involvements, which was found in 12% of patients, are cardiovascular complications, including the rise of troponin, myocarditis, blocks, arrhythmia, cardiac ischemia, heart failure, endocarditis, pericarditis and rarely reported tamponade. Compared to other cardiac complications, Myocardial injury prevalence is statistically dominant [5, 10, 11]. On the other hand, endocarditis has a tiny share. It has been rarely reported among several cardiac involvements of COVID-19 infection with different pathogeneses and prevalence [10, 12]. Direct action of the COVID-19 virus, the inflammatory phase of disease and cytokine storm, neurohumoral damage in the cardiovascular system, severe hypoxia, electrolyte abnormalities, increased shear stress and hypercoagulable state caused by COVID-19 infection are some of the hypothesised pathogenesis of cardiac complications following COVID-19 illness [3, 11, 13]. However, reported cases of infective endocarditis do not support direct impaction of the COVID-19 virus or inflammatory phase [14]. Previously reported cases raise different scenarios to explain the occurrence of endocarditis after COVID-19 infection. The first scenario is the primary presence of endocarditis, which is underdiagnosed because of the massive prevalence of COVID-19 infection and the pandemic. In this scenario, preexisting endocarditis can progress and even get worse because needed not only antibiotic administration delayed, but also immunocompromising agents are administered to treat COVID-19 infection, which can exacerbate the underlying disease [12, 15]. In these cases, the COVID-19 PCR test can be positive or not. Although in those patients with positive PCR tests and typical clinical findings and symptoms, COVID-19 infection cannot be ruled out; therefore, preexisting endocarditis is the actual cause of illness [16, 17]. In our cases, patients experienced an episode of symptom-free time, indicating that the main pathology was cleared and patients were cured. Then another episode of illness and somehow different signs and symptoms could be induced by relapsing the main pathogenesis or a new one. Therefore, our presented cases are not in favour of this hypothesis enough. The other scenario, supported by the presented cases in this article, is the probability of secondary infection after receiving immunocompromising agents to treat COVID-19 infection [18]. Corticosteroids as an anti-inflammatory agent are extensively used for an inflammatory phase of COVID-19 infection, which inhibits the immune system and will raise the possibility of secondary infection or deteriorating existing infection [19]: Tocilizumab, an interleukin 6 (IL6) receptor blocker. IL6 is a cytokine that is an essential component of the innate immune response, so blocking its action will compromise immune system function [8]. This can predispose secondary bacterial, viral, and fungal infections in patients theoretically, which is illustrated clinically in a few studies [8, 20–24]. However, primarily we should consider that several of these articles mentioned a short period of follow-up and challenging diagnosis due to mimicable symptoms and signs of COVID-19 infection as a limiting factor in the detection of secondary infections [21, 25–29]. Another hypothesis mentions critical illness and intensive unit care (ICU) admission contributing to secondary infection, pushing tocilizumab administration out of the spotlight [6]. Summing up the whole data, given that both cases presented in this article had corticosteroid and tocilizumab administration in their past medical history, the second hypothesis is highly supported in our patients of the published studies do not support this hypothesis clinically.

## Conclusion

Besides other mortal complications of COVID-19 infection, secondary infections following the administration of immunocompromising agents can result in acute diseases and conditions. We should be aware of these complications, including infective endocarditis, because early diagnosis and treatment can prevent morbidities and even mortality. However, further research is needed to prove the impact of tocilizumab administration on secondary infections after COVID-19 infection.

## Data Availability

The data sets used during the current study are available from the corresponding author upon reasonable request.

## Abbreviations

ARDS: Acute respiratory distress syndrome
PCR: Polymerase chain reaction
MRSA: Methicillin-resistant *Staphylococcus aureus*
MAS: Macrophage activating syndrome
NIH: National Institutes of Health
FDA: Food and Drug Administration
TPP: Time, place, and person
bpm: Beat per minute
mmHg: Millimeter of mercury
RV: Right ventricle
TR: Tricuspid regurgitation
TRG: Regurgitation gradient
PI: Pulmonary insufficiency
LV: Left ventricle
SPAP: Systolic pulmonary pressure
CT: Computed tomography
TVR: Tricuspid valve replacement
BD: Twice a day
DM: Diabetes mellitus
HTN: Hypertension
BMI: Body Mass Index
kg/m × m: Kilogram per mitre to the second power
MR: Mitral regurgitation
IAS: Inter atrial septum
MVR: Mitral valve replacement
PLHAMA: The patient left the hospital against medical advice
IL6: Interleukin 6
ICU: Intensive unit care

## References

[1] Inciardi RM, Lupi L, Zaccone G, Italia L, Raffo M, Tomasoni D (2020). Cardiac involvement in a patient with coronavirus disease 2019 (COVID-19). JAMA Cardiol.

[2] Siripanthong B, Nazarian S, Muser D, Deo R, Santangeli P, Khanji MY (2020). Recognising COVID-19–related myocarditis: the possible pathophysiology and proposed guideline for diagnosis and management. Heart Rhythm.

[3] Chimenti C, Magnocavallo M, Ballatore F, Bernardini F, Alfarano M, Della Rocca DG (2022). Prevalence and clinical implications of COVID-19 myocarditis. Cardiac Electrophysiol Clin.

[4] Buckley BJR, Harrison SL, Fazio-Eynullayeva E, Underhill P, Lane DA, Lip GYH (2021). Prevalence and clinical outcomes of myocarditis and pericarditis in 718,365 COVID-19 patients. Eur J Clin Invest.

[5] Parsova KE, Pay L, Oflu Y, Hacıyev R, Çinier G (2020). A rare presentation of a patient with COVID-19: cardiac tamponade. Turk Kardiyol Dern Ars.

[6] Tleyjeh IM, Kashour Z, Damlaj M, Riaz M, Tlayjeh H, Altannir M (2021). Efficacy and safety of tocilizumab in COVID-19 patients: a living systematic review and meta-analysis. Clin Microbiol Infect.

[7] Al-Baadani A, Eltayeb N, Alsufyani E, Albahrani S, Basheri S, Albayat H (2021). Efficacy of tocilizumab in patients with severe COVID-19: survival and clinical outcomes. J Infect Public Health.

[8] Antinori S, Bonazzetti C, Gubertini G, Capetti A, Pagani C, Morena V (2020). Tocilizumab for cytokine storm syndrome in COVID-19 pneumonia: an increased risk for candidemia?. Autoimmun Rev.

[9] Health NIo. COVID-19 Treatment Guidelines Panel [updated Monday, September 26, 2022. Available from: https://www.covid19treatmentguidelines.nih.gov/.

[10] Linschoten M, Peters S, van Smeden M, Jewbali LS, Schaap J, Siebelink HM (2020). Cardiac complications in patients hospitalised with COVID-19. Eur Heart J Acute Cardiovasc Care.

[11] Liu J, Virani SS, Alam M, Denktas AE, Hamzeh I, Khalid U (2021). Coronavirus disease-19 and cardiovascular disease: a risk factor or a risk marker?. Rev Med Virol.

[12] Alizadeh K, Bucke D, Khan S (2021). Complex case of COVID-19 and infective endocarditis. BMJ Case Rep.

[13] Kornowski R, Witberg G (2022). Acute myocarditis caused by COVID-19 disease and following COVID-19 vaccination. Open Heart..

[14] Amir M, Djaharuddin I, Sudharsono A, Ramadany S (2020). COVID-19 concomitant with infective endocarditis: a case report and review of management. Int J Infect Dis.

[15] Sengupta PP, Chandrashekhar YS (2020). Cardiac involvement in the COVID-19 pandemic: hazy lessons from cardiac imaging?. JACC Cardiovasc Imaging.

[16] Schizas N, Michailidis T, Samiotis I, Patris V, Papakonstantinou K, Argiriou M (2020). Delayed diagnosis and treatment of a critically ill patient with infective endocarditis due to a false-positive molecular diagnostic test for SARS-CoV-2. Am J Case Rep.

[17] Hayes DE, Rhee DW, Hisamoto K, Smith D, Ro R, Vainrib AF (2021). Two cases of acute endocarditis misdiagnosed as COVID-19 infection. Echocardiography.

[18] Ramos-Martínez A, Fernández-Cruz A, Domínguez F, Forteza A, Cobo M, Sánchez-Romero I (2020). Hospital-acquired infective endocarditis during Covid-19 pandemic. Infect Prev Pract.

[19] Gopalaswamy R, Subbian S (2021). Corticosteroids for COVID-19 therapy: potential implications on tuberculosis. Int J Mol Sci.

[20] Nguyen MT, Pødenphant J, Ravn P. Three cases of severely disseminated *Staphylococcus aureus* infection in patients treated with tocilizumab. BMJ Case Rep. 2013;2013. 10.1136/bcr-2012-007413 PMC3603831 23283607

[21] Zain Mushtaq M, Bin Zafar Mahmood S, Jamil B, Aziz A, Ali SA (2020). Outcome of COVID-19 patients with use of Tocilizumab: a single center experience. Int Immunopharmacol.

[22] Moreno-Pérez O, Andres M, Leon-Ramirez JM, Sánchez-Payá J, Rodríguez JC, Sánchez R (2020). Experience with tocilizumab in severe COVID-19 pneumonia after 80 days of follow-up: a retrospective cohort study. J Autoimmun.

[23] Celik I, Eryilmaz-Eren E, Kilinc-Toker A, Eren D, Yildiz M, Kanat A (2021). Low-dose tocilizumab is associated with improved outcome and a low risk of secondary infection in severe COVID-19 pneumonia. Int J Clin Pract.

[24] Sandhu G, Piraino ST, Piticaru J (2022). Secondary infection risk in patients with severe COVID-19 pneumonia treated with tocilizumab. Am J Ther.

[25] Aljuhani O, Al Sulaiman K, Alshabasy A, Eljaaly K, Al Shaya AI, Noureldeen H (2021). Association between tocilizumab and emerging multidrug-resistant organisms in critically ill patients with COVID-19: a multicenter, retrospective cohort study. BMC Infect Dis.

[26] Ruiz-Antorán B, Sancho-López A, Torres F, Moreno-Torres V, de Pablo-López I, García-López P (2021). Combination of tocilizumab and steroids to improve mortality in patients with severe COVID-19 infection: a Spanish, multicenter. Cohort Study Infect Dis Ther.

[27] Rojas-Marte G, Khalid M, Mukhtar O, Hashmi AT, Waheed MA, Ehrlich S (2020). Outcomes in patients with severe COVID-19 disease treated with tocilizumab: a case-controlled study. QJM.

[28] Sánchez-Montalvá A, Sellarés-Nadal J, Espinosa-Pereiro J, Fernández-Hidalgo N, Pérez-Hoyos S, Salvador F (2021). Early outcomes in adults hospitalised with severe SARS-CoV-2 infection receiving tocilizumab. Med Clin (Barc).

[29] Moore JL, Stroever SJ, Rondain PE, Scatena RN (2021). Incidence of secondary bacterial infections following utilization of tocilizumab for the treatment of COVID-19—a matched retrospective cohort study. J Glob Infect Dis.

